# Enhancing Concrete Durability and Strength with Fly Ash, Steel Slag, and Rice Husk Ash for Marine Environments

**DOI:** 10.3390/ma17123001

**Published:** 2024-06-19

**Authors:** Rodolfo Barragán-Ramírez, Andrés González-Hernández, Jorge Bautista-Ruiz, Michel Ospina, Willian Aperador Chaparro

**Affiliations:** 1 Ingeniería de Materiales Aplicados, Facultad de Ingeniería, Universidad Autónoma de Tamaulipas, Tampico 89109, Mexicoandres.gonz@uat.edu.mx (A.G.-H.); 2 Centro de Investigación de Materiales Cerámicos, Universidad Francisco de Paula Santander, San José de Cúcuta 540003, Colombia; 3 Casta Roja Agroindustrial S.A., Guayaquil 090109, Ecuador; michelospina@gmail.com; 4 Departamento of Engineering, Universidad Militar Nueva Granada, Bogotá 110111, Colombia; william.aperador@unimilitar.edu.co

**Keywords:** rice hush, alkaline activator, saline environments, class F fly ash, mechanical performance

## Abstract

The effect of an alternative source of silica, based on class F fly ash mixed with blast furnace slag and activated by rice husk ash (RHA), to produce concrete exposed to marine environments was evaluated. Four mixtures activated by the combination of 85% NaOH 14M + 15% RHA were manufactured to achieve a liquid/solid ratio of 0.20. Fly ash was incorporated into the steel slag mixture at addition percentages of 20, 40, 60, and 80%, and evaluated at 28, 900, and 1800 days for pore and chloride ion absorption. In general, including rice husk ash in the mixture of fly ash and steel slag significantly affected mechanical performance because it was possible to obtain concrete with high mechanical resistance. Concerning the durability evaluation, the effect of the activator generated by rice husk ash was observed, and the increase in steel slag added to the cementitious samples improved the capacity of the material to resist the penetration and diffusion of chloride ions.

## 1. Introduction

In building construction, economic cost is based on land, labor, and materials. In most cases, materials represent the majority of the investment [1,2]. In developing countries, there is a great variety of construction materials, including ceramics, wood, steel, stone, and earth, but cement as a binder represents the basis of most modern constructions, whether as part of their structure (concrete) or its coating (mortars) [3]. Cement has become practically indispensable due to its durability, accessibility, and resistance, but it is not considered an economic or ecological material [4]. Therefore, its production and consumption are unsustainable worldwide [5].

Today, Portland cement is one of the most commonly used materials in the world. It is produced in around 150 countries, mainly in Asia, Europe, and the Middle East [6]. Its universal use, relatively low cost (compared to other construction materials), potential for industrial production, incredible versatility, and the excellent results obtained have meant that this material has relegated all of its predecessors to oblivion or to minor uses [7].

The cement production industry uses more energy than the steel, chemical, oil, and paper industries. Cement companies consume 50% of all primary energy resources and are responsible for 53% of total CO_2_ emissions [8]. Cement production is involves the calcination of raw materials in a furnace until they are reduced to clinker powder, for which between 3.6 and 6 MJ of thermal energy is required, depending on the process used [9].

Regarding greenhouse gas emissions, the main contribution of the cement industry is the amount of CO_2_ released into the environment from the decomposition of limestone to form calcium oxide (CaO) and the burning of fuels through a rotary kiln used to obtain clinker or thermal power plants to generate the necessary electrical energy [10].

The synthesis of geopolymers is a promising and continuously evolving field with the potential to transform construction practices and move towards more sustainable and environmentally friendly approaches. The use of rice husk in geopolymer synthesis has been studied as a part of the quest for sustainable and efficient alternatives in the construction industry. This abundant and low-cost agricultural by-product has shown promise as a raw material for geopolymer production due to its rich composition of silica and alumina. These components are crucial for forming three-dimensional network structures in geopolymers, imparting strength and durability to the resulting material. Furthermore, using rice husk in geopolymer synthesis offers significant environmental advantages by reducing dependence on non-renewable resources and promoting the reuse of agricultural waste. A considerable number of publications report on the requirement to improve rice husk-based geopolymers to achieve desirable mechanical strengths, highlighting the importance and relevance of this research field [11,12,13].

Geopolymers, with their sustainability and durability profile, are emerging as the construction material of the future. Their low carbon footprint and superior strength promise more durable buildings and a cleaner planet [14]. “geopolymer” refers to amorphous aluminosilicate networks of Na^+^ or K^+^ [15], formed through alkaline activation, known as synthesis or polymerization. The reactive materials have a chemical composition rich in silica and alumina and contain high amounts of highly energetic solid phases, such as fly ash, steel slag, and rice husk ash [16]. Notably, the raw materials used to produce geopolymers are characterized by shallow calcium content. As the industry evolves, these innovative materials are poised to revolutionize how we build, offering greener and more efficient solutions for future generations [17].

The alkaline activator plays a crucial role in geopolymerization, as the type of alkali cation incorporated into this solution determines the final structure of the geopolymer [18]. It acts as a guide to direct and control the polycondensation capacity and structural growth as rapidly as possible. K^+^ contributes to a higher degree of condensation in the geopolymer than Na^+^ when both are incorporated under similar conditions [19]. This is attributed to the fact that the K^+^ metal ion has higher basicity, which allows for a superior dissolution rate and leads to greater strength development in geopolymers. The choice of which alkaline activator to use depends on the final application of the geopolymer and the type of primary sources to be utilized. On the other hand, Afsar Ali et al. [20] reported that using NaOH as an alkaline activator results in a higher degree of dissolution of natural minerals compared to the incorporation of KOH. Garcia et al. [21] concluded that the reaction kinetics are favored when the alkaline activator contains soluble silicates compared to solutions of alkaline hydroxides.

When establishing the problems of high economic and environmental costs of Portland cement, the use of industrial by-products with excellent availability, little use, and a high content of silicon and aluminous materials has been studied, which, in the presence of humidity, react chemically with hydroxide [22]. Calcium is used at room temperature to form compounds that have cementitious properties. Among these by-products, rocks of volcanic origin, fly ash generated from thermoelectric plants, blast furnace slag, baked clay, rice husk, corn leaf ash, and sugar cane bagasse ash are noteable [10].

This study aims to investigate the preparation and characterization of geopolymers incorporating fly ash, steel slag, and rice husk ash, focusing on their performance in marine environments. We hypothesized that this combination of materials will enhance the durability and strength of concrete in such environments. We identified a gap in the literature regarding the specific evaluation of the performance of geopolymers in marine environments and the combined use of these three industrial by-products. This study fills that gap by providing data and observations on how these materials affect the properties of concrete under marine conditions. The results confirm our hypothesis, demonstrating that including these industrial by-products effectively improves the durability and strength of concrete in marine environments.

## 2. Materials and Methods

### 2.1. Cementitious Materials

Three materials were used to form the samples to obtain the cementitious material. Table 1 presents the chemical compositions of the materials determined by X-ray fluorescence (XRF) spectroscopy. The data was analyzed using a Panalytical Epsilon 1^®^, an energy-dispersive X-ray Spectroscopy (EDS)-type instrument.

The distribution of particle sizes of the cementitious materials under study was determined via laser diffraction particle size analysis using a Mastersizer 3000+. This method enabled the characterization of particles in the samples by measuring the scattering of laser light, providing detailed information on particle size and distribution within the sample. The first material was low-calcium fly ash ASTM C618 [23] Class F with CaO < 10%, which originated from a thermoelectric industry, and its particle size was 27 μm on average. The second material was granulated blast furnace slag (GBFS) derived from steel processing by-products, with an average grain size of 30 μm. The third material, rice husk ash (RHA), was obtained through heat treatment in a spontaneous combustion oven, initially having an average particle size of 125 μm, which was subsequently reduced to approximately 26 μm through grinding processes.

### 2.2. Mortar Mixtures and Description of Tests

The samples to be analyzed were manufactured using different weight combinations of fly ash and blast furnace slag. Rice husk ashes were used as an activator. Four mixtures were activated using a combination of 85% NaOH 14M + 15% RHA, to achieve a liquid/solid ratio of 0.20. These ratios were determined based on the mixing proportions and the chemical composition of the raw materials and activators. Table 2 shows each studied sample’s nomenclature and the percentage composition of fly ash and granulated blast furnace slag. Table 3 details the quantities used for the mortar mixtures. The molar ratio parameters for the analyzed mixtures are recorded in Table 4.

The percentages of activated rice husk ash in combination with sodium hydroxide were calculated based on the amorphous or reactive content of rice husk ash. The sodium hydroxide solution was prepared under ambient conditions with a temperature of 25 °C and relative humidity of 63%, using distilled water to achieve the required concentration. The activating solutions (sodium hydroxide/rice husk ash) were premixed and stored under humidity and room temperature conditions for two days.

The identification and quantification of the content of the crystalline and amorphous phases in the cementitious mixtures was determined using an Empyrean Model X-ray Diffractometer. The diffractometer used monochromatic Co-Kα radiation with a wavelength of 1.78900 Å. It operated in a Bragg-Brentano geometry, with a goniometer in θ/θ configuration, covering a 2θ range between 10° and 90°. The step size and time were set at 0.02° and 0.2 s, respectively. This method is based on an interaction between X-rays and crystalline matter that produces diffraction. The samples were crushed very finely and mounted on a suitable support for the procedure. They were subsequently irradiated with X-rays of a specific wavelength, while the detector rotated simultaneously, allowing the crystalline planes to adjust to Bragg’s law and diffraction to occur.

The compressive strength of the concrete was determined at ages 28, 900, and 1800 days, according to the ASTM C192 standard [24]. The specimens were manufactured with cylindrical geometry, with dimensions of 0.20 m in height and 0.10 m in diameter. For the curing conditions, the specimens were immersed and air-dried. A hydraulic press was used to determine mechanical resistance. The rapid penetration of chloride ions was evaluated following the ASTM 1202 standard [25]. Using the ASTM 642 standard [26], the absorption and volume of permeable pores were determined.

Electrochemical impedance spectroscopy (EIS) assays were carried out using GAMRY 1010E TM interface electrochemistry equipment. The working electrode was ASTM A29 steel, the counter electrode was a graphite bar, and Ag/AgCl served as a reference electrode. The study involved exposure tests of reinforced concrete to a 3.5% NaCl saline solution over 1800 days. At specific exposure intervals of 28, 900, and 1800 days, the electrochemical behavior of the reinforcing steel in the concrete specimens was evaluated. These electrochemical tests provided crucial information about steel corrosion in concrete, a common issue in structures exposed to aggressive environments. The measurements covered frequency scanning from 100 kHz to 10 mHz. The sinusoidal voltage had an amplitude of 10 mV.

Each experiment was repeated three times using independent samples to minimize experimental error.

## 3. Results

**Figure 1:**
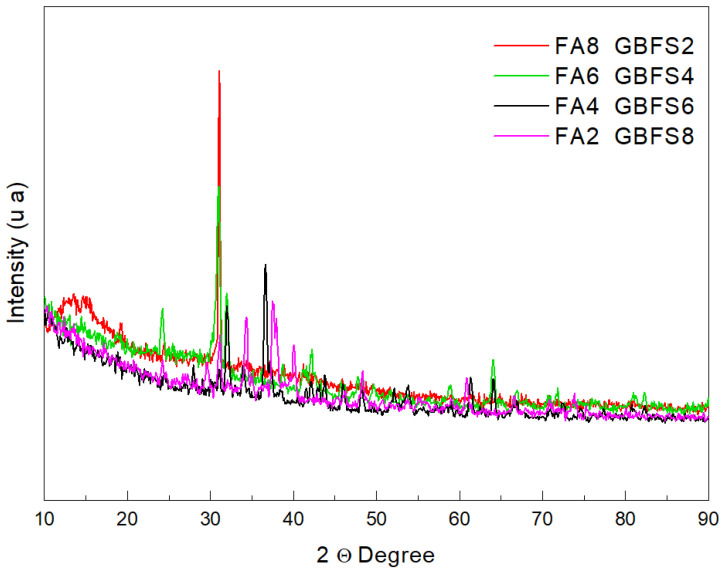
Diffractograms of the cementitious samples depending on the concentrations of by-products.

Figure 1 shows the diffraction patterns of the studied samples. In forming cementitious compounds based on pozzolanic materials, it is necessary to consider the amorphous silica content [27]. Table 5 shows the main crystalline compounds identified by X-ray diffraction [28]. The compounds are quartz (SiO_2_), mullite (Al_4_O_8_Si), calcite (CaCO_3_), graphite (C_4_), and nosean-carbonate (Na_8_Al_6_Si_6_O_24_-CO_3_). Other crystalline compounds found in a smaller proportion were aluminum-potassium silicate (Al_4_O_72.55_Si_32_-K_3.34_) with signals at 19.206° (2θ), ferrierite-K (Al_3_Si_3_O_11_-K) with signals at 53.92° (2θ), and microcline (AlSi_3_O_8_-K) with a signal at 30.387° (2θ) [29]. The signals of the compounds mentioned above appear in Figure 1, with greater intensity observed in the mixture with a higher fly ash content, especially the Microcline signal, which is also evident in the mixture of 80% ash—20% of scum.

Furthermore, for the mixture with a lower ash content, the nosean-carbonate and calcite signals were high-intensity and at an angle of 34.432° (2θ), and calcite was at an angle of 36.561° (2θ) [30]. In contrast, the calcite and eckermannite signals located at an angle of 37.537° (2θ) present low intensity.

**Figure 2:**
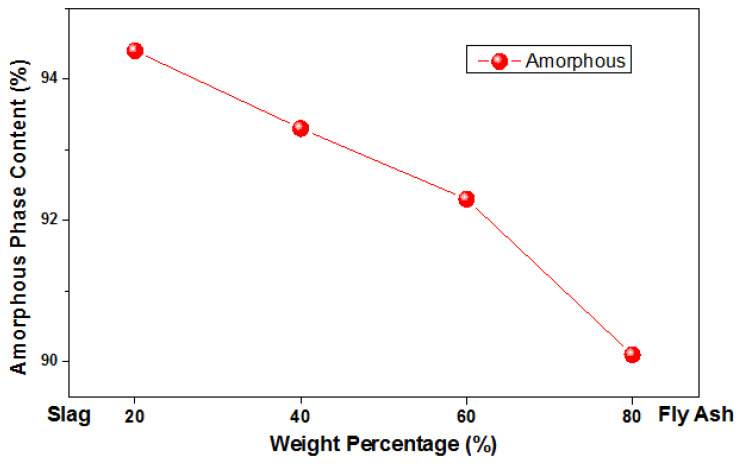
Variation of amorphous phase in fly ash and steel slag mixtures, activated with rice husk ash.

The K factor method was used to quantify the amorphous phase present in the mixtures. This method involved measuring an external crystalline standard to refine a calibration factor (K), which depended on the instrumental parameters and the characteristics of the incident beam. This method established that increasing the amount of fly ash in the mixture decreases the amount of amorphous material (by a small amount) and increases the amount of quartz and mullite, as seen in Table 5 and Figure 2.

These results were compared to other investigations in which diffraction patterns of the unmixed base materials containing significant amounts of amorphous material were obtained for the slag [31]. At the same time, predominant crystalline phases such as quartz and mullite were evident in the fly ash.

In the information recorded in Table 5, potassium alumino-silicate is observed in the samples with the lowest amount of slag due to the solid amorphous contribution [32]. Notably, the presence of potassium alumino-silicates in the mixture, due to the content of potassium oxides in the rice husk ash, favors the formation of lamellar compounds of the feldspar family [33]. The presence of these lamellar compounds could increase viscosity, improving the maneuverability of the mixtures.

**Figure 3:**
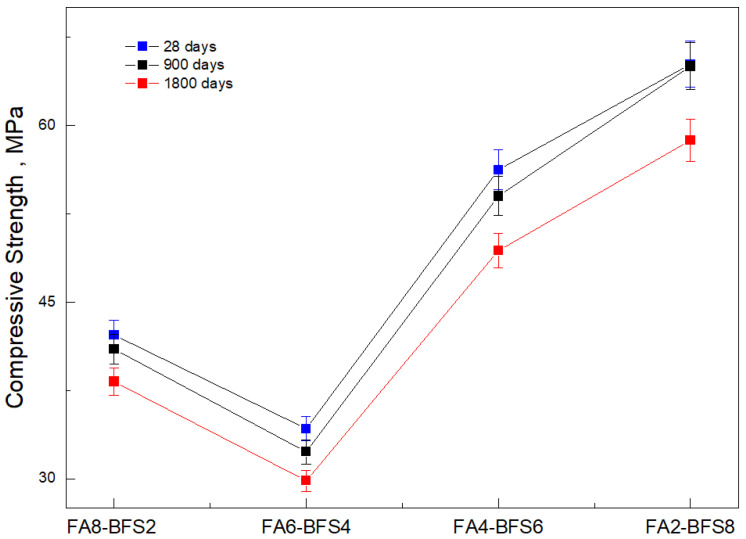
Diagram of compressive strength values of different mixtures and their behavior when immersed in a chloride ion solution.

Figure 3 shows the mechanical behavior of concrete with fly ash and steel slag additions, with varying addition percentages.

The compressive strength of the concrete was evaluated under exposure to chloride ions at curing times of 28 days, 900 days, and 1800 days [34]. The results established that the maximum resistance values are reached after 28 days of curing. In all mixtures, there was a tendency to reach the highest resistance values in the first days of curing, but these values varied depending on the concentrations of the additives [35]. It is essential to highlight that during the evaluation of the samples, the common factor was the immersion process in a solution composed of chloride ions. This condition significantly influenced the addition percentages without there being a relationship [36]. It is essential to highlight the results found for concrete samples with high percentages of fly ash, which presented the lowest resistance values. The unexpected decrease in mechanical properties upon reaching a 60% fly ash content could be explained by several factors. Firstly, at high levels of fly ash content, the effectiveness of the pozzolanic reaction may have decreased due to the saturation of fly ash particles in the concrete mix. This implies that a significant portion of the fly ash could have remained inactive, limiting its ability to strengthen the concrete by forming additional hydration products. Therefore, the 60% unreacted fly ash content is a filler in the matrix pores [37]. Lawrence et al. [38] also pointed out that adding fly ash could delay cement hydration, attributing this behavior to aluminate ions or dissolved organic material from the fly ash in the aqueous phase. Furthermore, Li et al. [39] stated that the degree of fly ash hydration was low in ternary mixtures with fly ash and slag additions, while slag produced better effects in strength development.

In concrete samples composed of 60% fly ash and 40% steel slag (FA6-GBFS4), evaluated after 28 days of curing, lower resistance values were obtained when subjected to a chloride ion attack [40]. According to Figure 3, all of the factors studied affect the behavior of compression resistance [41]. By varying the curing and interaction times with the chloride ion, the resistance of the concrete had an inverse performance to the test time. A significant effect on increasing porosity was also observed when adding more rice husk ash to the cementitious samples [42]. The FA6-GBFS4 mixture showed compressive strength values around 35 MPa, which was the lowest value. A linear trend of increase was evident with higher percentages of slag. The use of 80% blast furnace slag in the mixtures increased the compressive strength by approximately 40% compared to values in mixtures with 80% fly ash [43]. These results demonstrate a profitable effect on the compressive strength performance of cementitious samples due to blast furnace slag activation.

**Figure 4:**
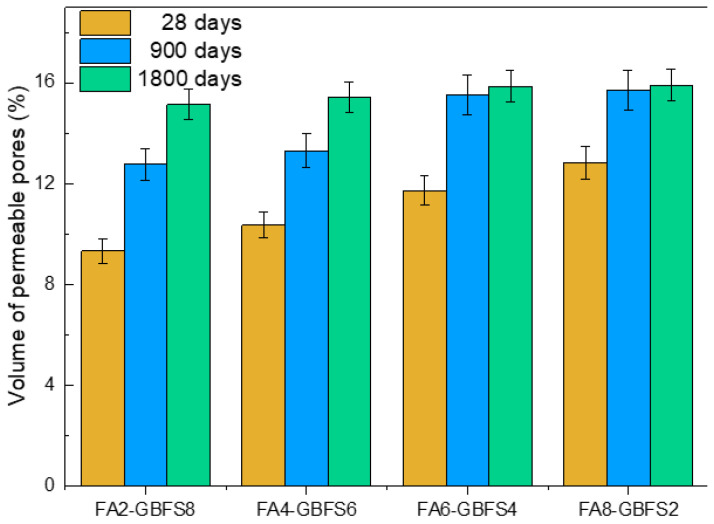
Permeable pore volume percentage depends on mixture type and evaluation time.

Figure 4 shows the effect of the volume of permeable pores for the addition percentages of fly ash and steel slag.

The significant effect of the activator was also evident, represented by a drastic change related to the increase in percentages close to 60% of fly ash addition [43]. Likewise, the chloride ion effect was harmful, since higher porosity values are reported when the samples are subjected to the solution. A proportional relationship was evident regarding greater pore volume at high fly ash additions. Furthermore, it was observed that the lowest percentages of porosity were obtained in the samples with the more significant addition of steel slag [44]. This performance enabled us to infer that the activation process due to slag improved the compressive strength and decreased the porosity percentages. For the set of samples studied, stability was observed in the percentage of porosity at 1800 days because, due to the entry of chloride ions into the samples, more pores were interconnected, resulting in less sealing of the cement matrix.

Regarding the interconnection of pores and its influence on the sealing of the cement matrix, the hydration of mineral additives is a complex process, as indicated by Wang and Lee [45]. They explained that fly ash, composed of aluminum and silica phases, lacks cementitious properties. However, when finely divided and in contact with moisture, fly ash undergoes a chemical reaction with the calcium hydroxide in cement to form cementitious compounds. Additionally, Gesoğlu and Özbay [46] pointed out that the pozzolanic reaction of fly ash in concrete depends on the breakdown and dissolution of the glassy phase, which occurs when the pH of the pore solution exceeds 13, suggesting a gradual reaction over time under appropriate alkaline conditions.

**Figure 5:**
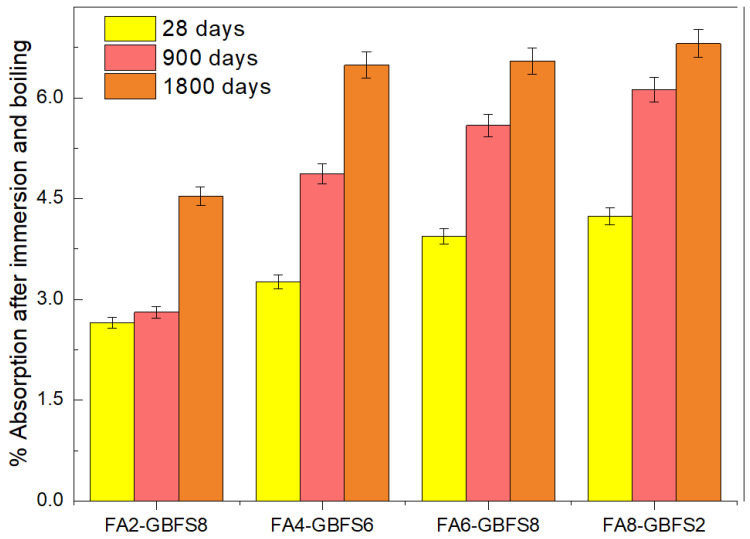
Percentage of chloride ion absorption depends on type of mixture and evaluation time.

Figure 5 shows the graph related to the absorption percentages of the samples studied.

Absorption is related to the movement of a fluid through the interconnections between pores [47]. The FA2-BFS8 mixture analyzed at curing times from 28 to 900 days showed the lowest absorption percentage values. However, at 1800 days, the absorption percentage doubled due to the greater interconnectivity between pores. There was no significant difference in the absorption percentages for samples FA4-GBFS6, FA6-GBFS4, and FA8-GBFS2 at 1800 days of curing.

Additionally, a proportional trend was observed between the percentage of absorption and the addition of fly ash in the cementitious mixtures. Figure 5 shows that the most influential factor in the absorption percentage is the exposure to the chloride ion. According to the figure, the longer the exposure time, the higher the absorption percentage values. This behavior is related to the movement produced in the capillaries of the concrete pores exposed to the fluid.

**Figure 6:**
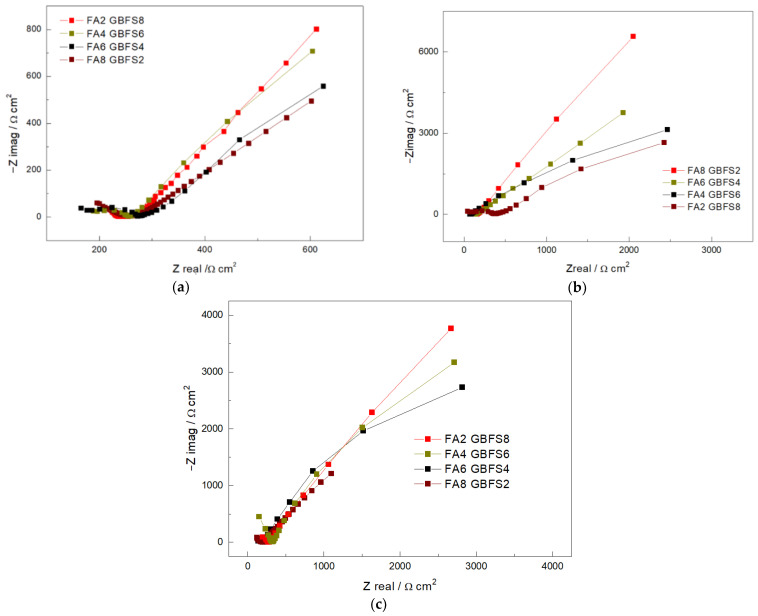
Nyquist diagrams of concrete mixtures activated with rice husk ash evaluated (**a**) 28 days, (**b**) 900 days, and (**c**) 1800 days.

Figure 6 shows the Nyquist diagrams of the concrete mixtures activated with rice husk ash at evaluation times of 28 days, 900 days, and 1800 days, respectively.

According to the results, it is evident that adding fly ash to the cement samples generated a shift to the right in the Nyquist diagrams. This displacement is related to the effect of fly ash on the resistance to degradation. The greater the amount of fly ash added, the lower the degradation resistance values. These results confirmed the behavior observed in the porosity and absorption tests, where the use of fly ash as a partial replacement of the mixture led to a lower resistivity of the concrete. It can be seen that all concrete mixtures demonstrate good resistance to polarization since their impedance is high in all of the cases evaluated, and that the concretes that have the highest content of steel slag are the ones that develop the highest impedance value. Although the porosity and absorption techniques show similar behaviors, to analyze the impact of the variables on the properties of concrete, the variations of the curves from the EIS technique were used. This made it possible to observe the differences over time, and its resistive and capacitive behavior.

In the case of the 80% steel slag mixture (FA2-GBFS8), concerning the 28-day reading, it was observed that the concrete did not generate protection. This behavior is similar to the other mixtures (Figure 6a). After 900 days (Figure 6b) from the first reading, an increase in impedance was observed, indicating resistance to degradation [48]. Increases in resistance to load transfer led to a matrix with low corrosion rates. The variable with the most influence on the response to an increase in impedance was the activator generated by the rice husk. The addition of steel slag to the cement samples improved the resistance to polarization [49].

Figure 6c shows the impedance spectrum and superposition of the conducts of the concrete mixtures at 1800 days of immersion in sodium chloride. This figure shows that high fly ash content in the samples improves durability. In this type of pozzolana, greater capacitance is observed due to the generation of a passive layer in the steel embedded in the concrete due to the hydration products generated by the mixture reactions. Additionally, it was found that, unlike at 900 days, the total impedance value was lower. This characteristic could be observed in more detail in the area closest to the axis of the impedance; however, the performance shifted to the left, which could be attributed to electrolyte and material resistance. The more the fluid in contact with the steel, the more the impedance stabilizes.

## 4. Conclusions

The incorporation of fly ash in the steel slag mixture, at addition percentages of 20, 40, 60, and 80% and evaluated at 28, 900, and 1800 days, revealed that compressive strength was more significant for the mixtures with a lower ratio-quantity of fly ash. However, in mixtures with 20% and 40% of fly ash, the resistance is adequate, and its values are similar. This is due to the morphological characteristics of fly ash related to porosity and surface area.

The percentage of addition in the mixtures at curing ages of 28 days is essential because mechanical compressive strengths higher than those in high-performance concrete are achieved. In general, the inclusion of rice husk ash in the mixture of fly ash and steel slag had a significant effect in terms of mechanical performance because it was possible to obtain concrete with high mechanical resistance, where factors such as the percentage of addition, particle size, crystal structure, and shell burning temperature play essential roles in obtaining a siliceous product suitable for these types of applications.

The electrochemical impedance spectroscopy tests determined that the passive condition of the reinforcing steel is achieved after 28 days of curing. After 900 days of evaluation in a fluid containing chloride ions, it was observed that a threshold value was reached due to transport phenomena in the porous medium. Subsequently, the steel depassivated, initiating corrosion. This was determined with the porosity and absorption tests, which allowed us to understand the transport mechanism of liquids and ions by various physical principles such as absorption and ion migration. Therefore, it was possible to determine, on a laboratory scale, the behavior of cementitious mixtures in the presence of agents that affect the passive condition of the reinforcing steel.

## Figures and Tables

**Table 1:** Chemical composition of cementitious materials used.

Chemical Composition (wt.%)			
Compound	FA%	GBFS%	RHA%
SiO_2_	54.30	33.70	90.93
Al_2_O_3_	28.8	12.80	0.11
Fe_2_O_3_	5.30	0.48	0.19
CaO	6.40	45.40	0.36
MgO	0.80	1.00	0.33
Na_2_O	0.90	0.12	0.02
K_2_O	0.70	1.50	1.97
P_2_O_5_	0.70	-	-
TiO_2_	1.20	0.50	-
MnO	0.01	-	-
SO_3_	0.92	-	0.15
SiO_2_/Al_2_O_3_	1.88	2.63	-
Unburnt	6.50	-	4.10

**Table 2:** Nomenclature and composition of samples.

Sample	FA (%)	GBFS (%)
FA8-GBFS2	80	20
FA6-GBFS4	60	40
FA4-GBFS6	40	60
FA2-GBFS8	20	80

**Table 3:** Proportions of mortar mixtures.

Ratio of Activator	Sample	FA	GBFS	Fine Aggregate	RHA	Coarse Aggregates	NaOH	Water Additional
		(kg/m^3^)						
85% NaOH 15% RHA	FA8-GBFS2	360	90	675	37	950	210	1
	FA6-GBFS4	270	180	675	37	950	210	5
	FA4-GBFS6	180	270	675	37	950	210	8
	FA2-GBFS8	90	360	675	37	950	210	11

**Table 4:** Molar ratios for mortar mixtures.

Molar Ratios					
Activator	Sample	SiO_2_/Al_2_O_3_	Na_2_O/Al_2_O_3_	H_2_O/Na_2_O	CaO/SiO_2_
85% NaOH 15% RHA	FA8-BFS2	4.48	1.84	4.18	0.22
	FA6-BFS4	4.54	2.00	4.32	0.43
	FA4-BFS6	4.62	2.19	4.45	0.69
	FA2-BFS8	4.70	2.43	4.47	0.98

**Table 5:** Crystalline compounds identified by X-ray diffraction.

Crystalline Phases (%)	Sample			
	FA8-GBFS2	FA6-GBFS4	FA4-GBFS6	FA2-GBFS8
Quartz (SiO_2_)	4.8	3.3	3	2.2
Mullite (Al_4_O_8_Si)	3.6	1.7	1.7	0.7
Calcite (CaCO_3_)	0.1	0.2	0.6	0.9
Akermatite (Ca_2_MgSi_2_O_7_)	0.1	0.1	0.3	0.4
Graphite (C_4_)	1.1	2.3	0.9	0.4
Nosean-Carbonate (Na_8_Al_6_Si_6_O_24_-CO_3_)	0.2	0.1	0.2	1
Total Crystalline Phases (%)	9.9	7.7	6.7	5.6

## Data Availability

The data that support the findings of this study are available from the first author.

## References

[1] Griffiths S., Sovacool B.K., Furszyfer Del Rio D.D., Foley A.M., Bazilian M.D., Kim J., Uratani J.M. (2023). Decarbonizing the cement and concrete industry: A systematic review of socio-technical systems, technological innovations, and policy options. Renew. Sustain. Energy Rev..

[2] Junaid M.F., Rehman Z., Kuruc M., Medveď I., Bačinskas D., Čurpek J., Čekon M., Ijaz N., Ansari W.S. (2022). Lightweight concrete from a perspective of sustainable reuse of waste by-products. Constr. Build. Mater..

[3] Sillanpää M., Ncibi C., Sillanpää M., Ncibi C. (2019). Circular economy in action: Case studies about the transition from the linear economy in the chemical, mining, textile, agriculture, and water treatment industries. The Circular Economy.

[4] Schneider M. (2019). The cement industry on the way to a low-carbon future. Cem. Concr. Res..

[5] Ramesh M., Palanikumar K., Hemachandra K. (2017). Plant fiber based bio-composites: Sustainable and renewable green materials. Renew. Sustain. Energy Rev..

[6] Imbabi M.S., Carrigan C., McKenna S. (2012). Trends and developments in green cement and concrete technology. Int. J. Sustain. Built Environ..

[7] Rissman J., Bataille C., Masanet E., Aden N., Morrow W.R., Zhou N., Elliott N., Dell R., Heeren N., Huckestein B. (2020). Technologies and policies to decarbonize global industry: Review and assessment of mitigation drivers through 2070. Appl. Energy.

[8] Ngab A.S., Zingoni A. (2001). Structural Engineering and Concrete Technology in Developing Countries: An Overview. Structural Engineering, Mechanics and Computation.

[9] Segura I.P., Ranjbar N., Damø A.J., Jensen L.S., Canut M., Jensen P.A. (2023). A review: Alkali-activated cement and concrete production technologies available in the industry. Heliyon.

[10] Jamora J.B., Go A.W., Gudia S.E.L., Giduquio M.B., Loretero M.E. (2023). Evaluating the use of rice residue ash in cement-based industries in the Philippines—Greenhouse gas reduction, transportation, and cost assessment. J. Clean. Prod..

[11] Poojalakshmi E.S., Patel J., Sunantha B., Thomas B.S., Ramaswamy K.P., Khan R.A. (2023). Effect of mechanical activation on the properties of rice husk ash-based one part geopolymer. Mater. Today Proc..

[12] Zhao Y., Chen B., Duan H. (2023). Effect of rice husk ash on properties of slag based geopolymer pastes. J. Build. Eng..

[13] Liang G., Zhu H., Li H., Liu T., Guo H. (2021). Comparative study on the effects of rice husk ash and silica fume on the freezing resistance of metakaolin-based geopolymer. Constr. Build. Mater..

[14] Moutaoukil G., Sobrados I., Alehyen S., Taibi M. (2024). Monitoring the Geopolymerization Reaction of Geopolymer Foams Using 29Si and 27Al MAS NMR. Minerals.

[15] Alouani M.E., Saufi H., Aouan B., Bassam R., Alehyen S., Rachdi Y., Hadki H.E., Hadki A.E., Mabrouki J., Belaaouad S. (2024). A comprehensive review of synthesis, characterization, and applications of aluminosilicate materials-based geopolymer. Environ. Adv..

[16] Siyal A.A., Mohamed R.M.S., Shamsuddin R., Ridzuan M.B. (2024). A comprehensive review of synthesis kinetics and formation mechanism of geopolymers. RSC Adv..

[17] Li C., Fu Y., Cheng H., Wang Y., Jia D., Liu H. (2024). Green and Low-Cost Modified Pisha Sandstone Geopolymer Gel Materials for Ecological Restoration: A Phase Review. Gels.

[18] Martínez-Martínez S., Bouguermouh K., Bouzidi N., Mahtout L., Sánchez-Soto P.J., Pérez-Villarejo L. (2024). Preparation of Geopolymeric Materials from Industrial Kaolins, with Variable Kaolinite Content and Alkali Silicates Precursors. Materials.

[19] Davidovits J. (1976). Solid phase synthesis of a mineral blockpolymer by low temperature polycondensation of aluminosilicate polymers. IUPAC International Symposium on Macromolecules, Topic III, New Polymers of High Stability.

[20] Ali A., Khan Q., Mehboob S.S., Tayyab A., Hayyat K., Khan D., Haq I.U., Qureshi Q. (2024). Enhancing multi-objective mix design for GGBS-based geopolymer concrete with natural mineral blends under ambient curing: A Taguchi-Grey relational optimization. Ain Shams Eng. J..

[21] García-Lodeiro I., Fernández-Jiménez A., Palomo A., Pacheco-Torgal F., Jalali S., Labrincha J., John V.M. (2013). 17—Alkali-activated based concrete. Woodhead Publishing Series in Civil and Structural Engineering, Eco-Efficient Concrete.

[22] Antzaras A.N., Papalas T., Heracleous E., Kouris C. (2023). Techno–economic and environmental assessment of CO_2_ capture technologies in the cement industry. J. Clean. Prod..

[23] (2022). Standard Specification for Coal Fly Ash and Raw or Calcined Natural Pozzolan for Use in Concrete.

[24] (2014). Standard Practice for Making and Curing Concrete Test Specimens in the Laboratory.

[25] (2019). Standard Test Method for Electrical Indication of Concrete’s Ability to Resist Chloride Ion Penetration.

[26] (2021). Standard Test Method for Density, Absorption, and Voids in Hardened Concrete.

[27] Navarrete I., Valdes J., Lopez M., Vargas F. (2023). Replacement of pozzolanic blended cement by supplementary cementitious materials: Mechanical and environmental approach. Constr. Build. Mater..

[28] Bonifacio A.L., Archbold P. (2022). The effect of calcination conditions on oat husk ash pozzolanic activity. Mater. Today Proc..

[29] Wang C., Chen S., Huang D., Chen Q., Tu M., Wu K., Zhang Z. (2022). Pozzolanic activity and environmental risk assessment of water-based drilling cuttings of shale gas. Constr. Build. Mater..

[30] Kang S., Kang H., Lee N., Kwon Y., Moon J. (2022). Development of cementless ultra-high performance fly ash composite (UHPFC) using nucleated pozzolanic reaction of low Ca fly ash. Cem. Concr. Compos..

[31] Patangia J., Saravanan T.J., Kabeer K.I., Bisht K. (2023). Study on the utilization of red mud (bauxite waste) as a supplementary cementitious material: Pathway to attaining sustainable development goals. Constr. Build. Mater..

[32] Wang F., Long G., Zhou J.L. (2023). Deep insight into green remediation and hazard-free disposal of electrolytic manganese residue-based cementitious material. Sci. Total Environ..

[33] Tokareva A., Kaassamani S., Waldmann D. (2023). Fine demolition wastes as Supplementary cementitious materials for CO_2_ reduced cement production. Constr. Build. Mater..

[34] Song B., Liu S., Hu X., Ouyang K., Li G., Shi C. (2022). Compressive strength, water and chloride transport properties of early CO_2_-cured Portland cement-fly ash-slag ternary mortars. Cem. Concr. Compos..

[35] Laxmi G., Patil S., Hossiney N., Thejas H.K. (2023). Effect of hooked end steel fibers on strength and durability properties of ambient cured geopolymer concrete. Case Stud. Constr. Mater..

[36] Alyousef R., Abbass W., Aslam F., Gillani S.A.A. (2023). Characterization of high-performance concrete using limestone powder and supplementary fillers in binary and ternary blends under different curing regimes. Case Stud. Constr. Mater..

[37] Jeong Y., Park H., Jun Y., Jeong J.H., Oh J.E. (2015). Microstructural verification of the strength performance of ternary blended cement systems with high volumes of fly ash and GGBFS. Constr. Build. Mater..

[38] Lawrence P., Cyr M., Ringot E. (2003). Mineral admixtures in mortars: Effect of inert materials on short-term hydration. Cem. Concr. Res..

[39] Li D., Shen J., Chen Y., Cheng L., Wu X. (2000). Study of properties of fly ash–slag complex cement. Cem. Concr. Res..

[40] Klemczak B., Gołaszewski J., Smolana A., Gołaszewska M., Cygan G. (2023). Shrinkage behaviour of self-compacting concrete with a high volume of fly ash and slag experimental tests and analytical assessment. Constr. Build. Mater..

[41] Nakum A.V., Arora N.K. (2023). Fresh and mechanical characterization of fly ash/slag by incorporating steel fiber in self-compacted geopolymer concrete. Constr. Build. Mater..

[42] Kuranlı Ö.F., Uysal M., Abbas M.T., Cosgun T., Niş A., Aygörmez Y., Canpolat O., Al-mashhadani M.M. (2022). Evaluation of slag/fly ash based geopolymer concrete with steel, polypropylene and polyamide fibers. Constr. Build. Mater..

[43] Mocharla I.R., Selvam R., Govindaraj V., Muthu M. (2022). Performance and life-cycle assessment of high-volume fly ash concrete mixes containing steel slag sand. Constr. Build. Mater..

[44] Li L., Ye T., Li Y., Wang Z., Feng Z., Liu Z. (2023). Improvement of microporous structure and impermeability of cement mortars using fly ash and blast furnace slag under low curing pressures. Constr. Build. Mater..

[45] Wang X.Y., Lee H.S. (2010). Modeling the hydration of concrete incorporating fly ash or slag. Cem. Concr. Res..

[46] Gesoğlu M., Özbay E. (2007). Effects of mineral admixtures on fresh and hardened properties of self-compacting concretes: Binary, ternary and quaternary systems. Mater. Struct..

[47] Aperador W., Mejía de Gutiérrez R., Bastidas D.M. (2009). Steel corrosion behaviour in carbonated alkali-activated slag concrete. Corros. Sci..

[48] Huo Y., Huang J., Lu D., Han X., Sun H., Liu T., Wang J., Wang F., Tan P., Wang M. (2023). Durability of alkali-activated slag concrete incorporating silica fume and rice husk ash. J. Build. Eng..

[49] Abbass M., Singh G. (2022). Durability of rice husk ash and basalt fiber based sustainable geopolymer concrete in rigid pavements. Mater. Today Proc..

